# Modification of Polyhedral Oligomeric Silsesquioxanes (POSS) Molecules by Ruthenium Catalyzed Cross Metathesis

**DOI:** 10.3390/molecules23071722

**Published:** 2018-07-14

**Authors:** Justyna Czaban-Jóźwiak, Łukasz Woźniak, Artur Ulikowski, Katarzyna Kwiecińska, Adam A. Rajkiewicz, Karol Grela

**Affiliations:** Institute of Organic Chemistry, Polish Academy of Sciences, Kasprzaka 44/52, PO Box 58, 01-224 Warsaw, Poland; lukasz.wozniak5@gmail.com (Ł.W.); artur.ulikowski@gmail.com (A.U.); kwiecinskaxyz@gmail.com (K.K.); a.rajkiewicz@cent.uw.edu.pl (A.A.R.)

**Keywords:** cross metathesis, cross metathesis (CM), steroid, POSS, polyhedral oligomeric silsesquioxanes, ruthenium complexes

## Abstract

The scope of ruthenium (Ru)-catalyzed cross metathesis (CM) of allyl-decorated polyhedral oligomeric silsesquioxanes (POSS) was explored. A variety of different commercial and non-commercial ruthenium complexes were tested to determine that the nitro-activated Ru catalyst is optimal for this transformation. The reported transformation was used to prepare selected hybrid steroid-POSS compounds.

## 1. Introduction

**Figure 1:**
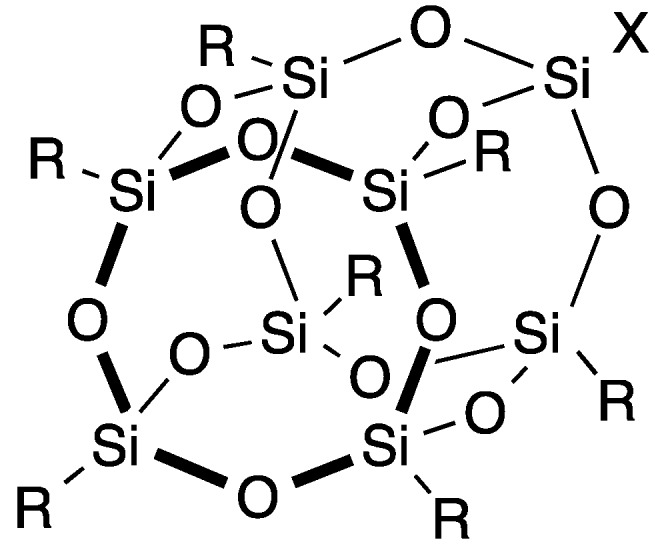
The general features of polyhedral oligomeric silsesquioxanes (POSS).

Polyhedral oligomeric silsesquioxanes (POSS) are characterized by their remarkable cubic structure, which has received attention from scientists of different disciplines [1,2,3]. POSS can be used as precursors and components in a variety of hybrid materials for biomedical applications, such as biomedical devices, tissue engineering scaffolds, drug delivery systems, dental applications, and biological sensors [4,5,6,7,8,9,10]. The POSS molecule (Figure 1) consists of a cubic silica-oxygen core that is surrounded by eight tunable groups, one in each corner of the cube. These groups can be identical or one of them can be different, allowing for further site selective modification. The chain groups (R, X) can be substituted with a potentially unlimited number of organofunctional groups, such as alkyls, olefins, alcohols, esters, anhydrides, amines, imides, epoxides, thiols, sulfonates, silanols, and many others [11].

Olefin cross metathesis (CM) is one of the most powerful methods for preparing variously substituted alkenes [12,13,14,15]. CM is widely used as a key step in the synthesis of many chemicals from simple oils [16,17] to complex structures that exhibit biological activity [18,19,20].

**Figure 2:**
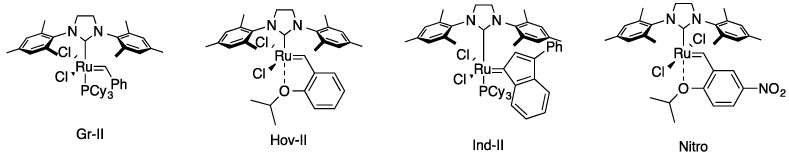
Commonly used types of ruthenium catalysts for olefin metathesis.

CM is catalyzed by ruthenium (Ru) or molybdenum (Mo) complexes; the most common catalysts are ruthenium complexes such as Grubbs (**Gr**), Hoveyda-Grubbs (**Hov**) and Indenylidene (**Ind**) (Figure 2) [21].

The first cross metathesis reaction of octavinyl POSS was performed by the Feher group in 1997 employing Schrock and Grubbs catalysts [22]. Schrock catalysts were substantially more active compared to Grubbs. The authors showed that metathesis offers an important new route in the functionalization of POSS derivatives. Even though Schrock catalysts provided better results, the authors emphasized that ruthenium catalysts are the future of POSS CM. Marciniec et al. reported the first efficient CM of vinyl-POSS in the presence of a ruthenium catalyst [23,24,25]. POSS CM was also applied for the synthesis of POSS-core dendrimers [26,27]. Notably, this methodology required extremely high catalyst loading (45 mol%) of first generation Grubbs catalyst (**Gr-I**) [26,27].

The CM reaction of POSS derivatives might be considered the most direct method for obtaining new POSS biohybrids, which is being intensively explored in nanomedicine [4]. Herein, we report the first examples of allyl-POSS CM reaction. To achieve this transformation, a variety of commercially available ruthenium complexes, as well as a few obtained in our laboratories, were screened in two model CM reactions of allyl-POSS derivative (**1**), using easily reacting *cis*-1,4-Diacetoxy-2-butene (**3**) and the more challenging, electron-deficient, *tert*-butyl acrylate (**2**) as CM partners. After selecting the most efficient catalysts the utility of this method was demonstrated by the synthesis of some selected POSS-steroid conjugates.

## 2. Results and Discussion

The first step in the present study was to choose an appropriate catalyst and conditions. For this approach, we selected *tert*-butyl acrylate (**2**) and (*Z*)-diacetoxy-but-2-en (**3**) as the model CM partners (Table 1). The CM reactions were performed at room temperature in dichloromethane (DCM). Notably, the catalyst loading in these preliminary experiments was set at 0.5 mol%, which is substantially lower than in the previous reports on CM of POSS molecules of 45 mol%.

**Figure 3:**
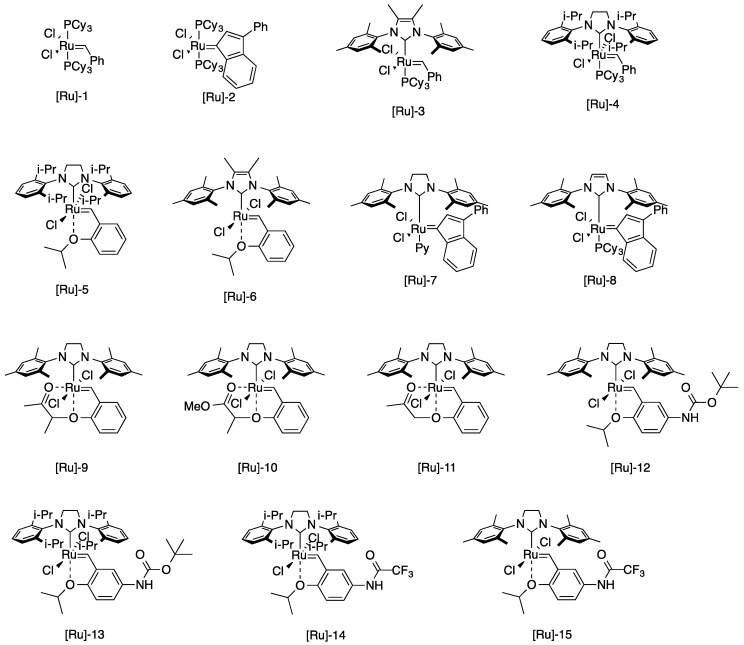
Additional ruthenium complexes used in this study.

Almost all tested complexes (Figure 2 and Figure 3) presented some activity toward POSS CM. First generation catalysts **[Ru]-1** and **[Ru]-2** (Table 1, Entry 1 and 2, respectively) showed the lowest activity among the catalysts studied here. In the Indenylidene family, complexes **Ind-2** (Table 1, Entry 10) and **[Ru]-8** (Table 1, Entry 12) did not catalyse the process, whereas **[Ru]-7** (Table 1, Entry 11) exhibited medium activity. However, those complexes perform better at elevated temperatures [28]. Complexes with SIPr N-Heterocyclic Carbene (NHC) ligands **[Ru]-4** (Table 1, Entry 5), **[Ru]-5** (Table 1, Entry 8), **[Ru]-13** (Table 1, Entry 22), and **Ru-[14]** (Table 1, Entry 23) displayed higher catalytic activity toward the CM reaction in comparison to the parent catalysts [20,29]. Complexes bearing so-called “Turbo” unsaturated NHC ligand **[Ru]-3** (Table 1, Entry 4) and **[Ru]-6** (Table 1, Entry 9) according the literature supposed to behave similarly to SIPr-modified complexes [30]. Interestingly, in this case, we observed lower catalytic activity. A modification of the benzylidene ligand, as in the case of **Nitro** (Table 1, Entry 13), **[Ru]-13** (Table 1, Entry 21), **[Ru]-14** (Table 1, Entry 23), and **[Ru]-15 [31]** (Table 1, Entry 25), did not improve catalytic activity compared to parent **Hov-II** (Table 1, Entry 10). Similarly, the additional chelation present in the case of complexes **[Ru]-9** (Table 1, Entry 15), **[Ru]-10 [32,33]** (Table 1, Entry 17), and **[Ru]-11** (Table 1, Entry 19) did not significantly change the reaction outcome.

**Figure 4:**
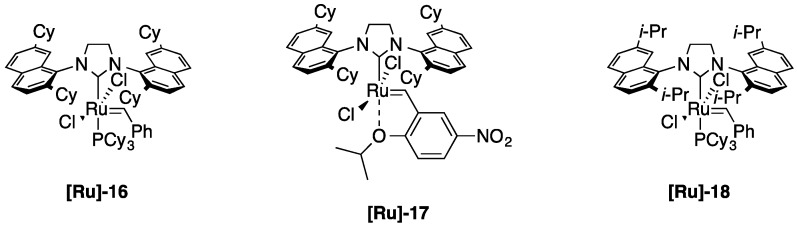
Family of ruthenium complexes bearing N-Heterocyclic Carbene (NHC) ligand with naphthyl side chains used in this study.

Next, we investigated the catalytic activity of Ru complexes bearing the NHC ligand with a more bulky naphthyl side chains (Dorta’s NHC ligands, Figure 4) [34]. This type of complexes possesses higher activity in some olefin metathesis transformations [35,36,37]. However, in the case of POSS CM, catalysts **[Ru]-16** (Table 1, Entry 26), **[Ru]-17** (Table 1, Entry 27), and **[Ru]-18** (Table 1, Entry 28) generally displayed similar activity as the other “decorated” Hoveyda-type complexes.

For further investigation of POSS metathesis with more sophisticated CM partners, the most popular **Hov-II** catalyst was selected due to its commercial availability and satisfying catalytic activity. Additionally, the **Nitro** catalyst was selected due to its high catalytic activity toward steroids [38] as well as its commercial availability.

As these steroidal CM partners can be more challenging in terms of reactivity than a simple diacetate **3** or even acrylate **2**, we expected that an increase in the reaction temperature and catalyst loading might be necessary to obtain practically useful product yields.

Reacting androsterone derivative **6** with allyl-POSS (**1**) (Table 2) in the previously determined optimal conditions yielded only traces (3%) of the desired product. Increasing the temperature to 45 °C and the loading of **Hov-II** to 2 mol% increased the yield of **7** to 36%. Further improvement was achieved by performing the reaction 100 °C in toluene to access the desired product **7** in 69% yield. Finally, when we performed the reaction in the presence of the **Nitro** catalyst, product **7** was obtained with 72% of yield as a mixture of Z/E isomers 20:80. 

Pregnenolone derivative **8** (Table 3) react with allyl-POSS to give the functionalized POSS **9** in 29% yield using 0.5 mol% of **Hov-II** at room temperature. Increasing the temperature to 100 °C did not significantly improve the yield (36%), as well as applying **Nitro** as a catalyst (46%)**.** However, the best result was obtained refluxing the reaction mixture in DCM for 24 h using 2 mol% of **Nitro** catalyst. In these conditions, the desired product **9** was obtained in 69% yield as a mixture of Z/E isomers 20:80

We were intrigued by very similar results obtained at room temperature and 100 °C using substrate **8**. We hypothesized that the higher reaction temperature might facilitate both reaction between and **1** and **8** but also self CM of **1**. We performed control experiments by mixing **1** with 0.5 mol% of **Hov-II** in toluene at 100 °C and in DCM at room temperature. Indeed, the experiment at elevated temperature resulted in 90% of the self cross metathesis product and no conversion was observed at room temperature. 

**Scheme 1:**
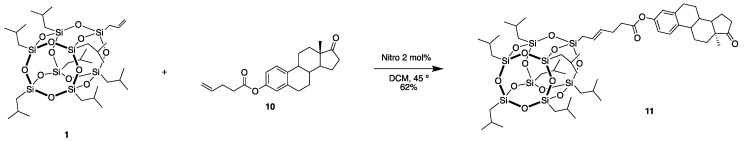
Cross metathesis of allyl-POSS **1** with the derivative of estrone **10**.

Next, we employed the optimal conditions for substrate **8** to prepare estrone POSS derivative **11** (Scheme 1) that was obtained as 20:80 mixture of Z/E isomers in 62% yield. 

## 3. Materials and Methods 

### 3.1. General 

Nuclear magnetic resonance (NMR) spectra were recorded in CDCl_3_ (chloroform-d) or benzene-d_6_ solutions (unless indicated otherwise); chemical shifts are reported in the δ scale in ppm, with the solvent signal as the internal standard (CDCl_3_, ^1^H NMR 7.26 ppm; ^13^C NMR 77.00 ppm, benzene-d_6_, ^1^H NMR 7.16 ppm, ^13^C NMR 128.06 ppm). Column chromatography was performed on Merck silica gel 60, 230–400 mesh (Darmstadt, Germany). Thin layer chromatography (TLC) was performed on aluminum sheets, Merck 60 F_254_ (Darmstadt, Germany). Anhydrous solvents were obtained by distillation over calcium chloride (CaCl_2_) (DCM, Toluene). All reactions were performed under argon (Ar) in pre-dried glassware using Schlenk techniques.

### 3.2. General Procedure for the Cross Metathesis of **1** with Different Partners

The catalyst was added in one portion to a solution of propyl-POSS and appropriate partner in DCM/Toluene (4 mL). The resulting mixture was stirred in an appropriate temperature under an argon atmosphere. The solvent was removed under reduced pressure. The crude product was purified by flash chromatography (c-hexane/EtOAc) to obtain pure product.

### 3.3. 4-(POSS)but-2-en-1-yl acetate (**4**)

^1^H NMR (500 MHz, CDCl_3_): δ, 5.79–5.70 (m, 1H), 5.66 (dtt, *J* = 11.4, 8.6, 1.4 Hz, 1H), 5.55–5.45 (m, 1H), 4.61 (dd, *J* = 6.8, 1.4 Hz, 1H), 4.48 (dd, *J* = 6.7, 1.1 Hz, 2H), 2.06 (s, 1H), 2.04 (s, 2H), 1.85 (dpd, *J* = 13.4, 6.7, 2.3 Hz, 7H), 1.66 (dd, *J* = 8.6, 1.4 Hz, 1H), 1.61 (dd, *J* = 7.9, 1.3 Hz, 2H), 0.96 (dd, *J* = 6.6, 1.0 Hz, 42H), 0.63–0.58 (m, 14H) ppm. ^13^C NMR (126 MHz, CDCl_3_): δ, 171.1, 171.0, 130.7, 128.6, 124.2, 122.9, 77.4, 77.3, 77.1, 76.9, 65.6, 60.6, 25.9, 25.8, 25.8, 24.0, 22.9, 22.6, 22.5, 21.2, 18.4, 14.6, 1.2, 0.1 ppm (Supplementary Materials). **IR** (film, DCM) ν 2954, 2927, 2907, 2870, 1745, 1465, 1401, 1383, 1366, 1332, 1230, 1169, 1107, 1038, 964, 838. **EA** Anal. Calcd. For C_34_H_72_O_14_Si_8_: C, 43.93; H, 7.81; found C, 43.77; H, 7.98. **MS (ESI)**: *m/z* [M + Na]^+^: 951.

### 3.4. tert-butyl 4-(POSS)but-2-enoate (**5**)

^1^H NMR (500 MHz, CDCl_3_): δ, 6.86 (dt, *J* = 15.4, 8.5 Hz, 1H), 5.67 (dt, *J* = 15.4, 1.4 Hz, 1H), 1.85 (dpd, *J* = 13.5, 6.8, 1.5 Hz, 7H), 1.75 (dd, *J* = 8.5, 1.4 Hz, 2H), 1.47 (s, 9H), 0.95 (dd, *J* = 6.6, 1.0 Hz, 43H), 0.63–0.58 (m, 14H) ppm. ^13^C NMR (126 MHz, CDCl_3_): δ, 166.1, 143.0, 123.3, 79.8, 79.7, 28.5, 28.4, 25.9, 25.8, 24.1, 24.0, 24.0, 22.7, 22.6, 22.5, 22.2, 22.1, 19.4, 1.2, 0.2 ppm (Supplementary Materials). **IR** (film, DCM) ν 2955, 2932, 2907, 2870, 1714, 1646, 1465, 1401, 1383, 1367, 1326, 1296, 1230, 1213, 1168, 1106, 1040, 982, 839. **EA** Anal. Calcd. For C_36_H_76_O_14_Si_8_: C, 45.15; H, 8.00; found C, 45.03; H, 7.93. MS (ESI): *m/z* [M + Na]^+^: 979.

### 3.5. (10R,13S)-10,13-dimethyl-1,2,3,4,7,8,9,10,11,12,13,14,15,16-tetradecahydrospiro[cyclopenta[a] phenanthrene-17,2’-[1,3]dioxolan]-3-yl 6-(POSS)hex-4-enoate (7)

^1^H NMR (400 MHz, C_6_D_6_): δ, 5.76–5.62 (m, 1H), 5.59–5.44 (m, 1H), 5.35–5.26 (m, 1H), 4.87 (ddt, *J* = 11.1, 8.4, 5.5 Hz, 1H), 3.61–3.46 (m, 4H), 2.58–2.46 (m, 1H), 2.41 (t, *J* = 6.9 Hz, 2H), 2.36–2.30 (m, 1H), 2.18–1.98 (m, 9H), 1.88 (dddd, *J* = 17.2, 14.9, 7.3, 4.0 Hz, 4H), 1.80–1.69 (m, 3H), 1.67–1.53 (m, 5H), 1.46 (dd, *J* = 11.4, 3.2 Hz, 3H), 1.36 (td, *J* = 13.8, 13.0, 5.0 Hz, 3H), 1.16–1.04 (m, 47H), 0.96 (d, *J* = 1.6 Hz, 3H), 0.91 (t, *J* = 2.3 Hz, 3H), 0.88–0.80 (m, 14H) ppm. ^13^C NMR (101 MHz, CDCl_3_): δ, 171.9, 139.8, 129.8, 124.6, 122.7, 119.6, 73.8, 65.2, 64.6, 50.9, 50.3, 46.1, 38.8, 37.2, 36.9, 34.9, 34.7, 32.4, 31.7, 30.9, 28.8, 28.3, 25.9, 24.5, 23.2, 23.1, 22.9, 20.9, 19.4, 18.5, 14.6 ppm (Supplementary Materials). **IR** (film, DCM) ν 3444, 2925, 2856, 1733, 1671, 1462, 1378, 1307, 1255, 1228, 1170, 1109, 1040, 959, 903, 881.

### 3.6. (13S)-13-methyl-17-oxo-7,8,9,11,12,13,14,15,16,17-decahydro-6H-cyclopenta[a] phenanthren-3-yl-6-(POSS)hex-4-enoate (**9**)

^1^H NMR (400 MHz, C_6_D_6_): δ, 5.94–5.82 (m, 0.2 × 1H), 5.76–5.65 (m, 1H), 5.62–5.52 (m, 0.8 × 1H), 5.35 (dt, *J* = 5.6, 1.8 Hz, 1H), 3.45–3.34 (m, 1H), 2.30–2.21 (m, 2H), 2.20–2.03 (m, 9H), 1.99–1.87 (m, 2H), 1.86–1.77 (m, 2H), 1.75–1.65 (m, 3H), 1.63–1.53 (m, 2H), 1.50–1.36 (m, 5H), 1.31 (s, 3H), 1.14–1.06 (m, 44H), 0.96 (s, 3H), 0.93 (s, 3H), 0.89–0.83 (m, 14H) ppm. ^13^C NMR (101 MHz, C_6_D_6_): δ, 141.3, 126.8, 125.6, 121.6, 74.9, 74.8, 74.3, 71.7, 59.2, 58.9, 58.8, 57.3, 50.6, 50.6, 47.6, 43.3, 43.1, 43.1, 42.9, 41.4, 40.7, 40.6, 37.7, 36.8, 32.3, 32.2, 31.7, 27.8, 27.2, 27.0, 26.0, 26.0, 25.9, 24.5, 24.4, 24.3, 24.2, 23.1, 23.0, 22.8, 22.5, 21.4, 21.4, 19.5, 18.6, 14.5, 14.0, 13.9, 13.8 ppm (Supplementary Materials). **IR** (film, DCM) ν 3387, 2954, 2871, 1708, 1465, 1401, 1382, 1366, 1333, 1229, 1110, 954, 837. MS (TOF ES): *m/z* [M + Na]^+^: 1210.53

### 3.7. ((13S,17R)-13-methyl-17-(4-(POSS)but-2-en-1-yl)-7,8,9,11,12,13,14,15,16,17-decahydro-6H-cyclopenta[a]phenanthrene-3,17-diol (**11**)

^1^H NMR (400 MHz, CDCl_3_): δ, 7.30–7.26 (m, 1H), 6.88–6.77 (m, 2H), 5.69–5.56 (m, 0.2 × 1H), 5.63–5.55 (m, 0.2 × 1H), 5.54–5.45 (m, 0.8 × 1H), 5.44–5.35 (m, 0.8 × 1H), 2.96–2.84 (m, 2H), 2.62–2.54 (m, 2H), 2.53–2.46 (m, 1H), 2.46–2.36 (m, 2H), 2.29 (t, *J* = 10.9 Hz, 1H), 2.21–2.07 (m, 1H), 2.07–1.93 (m, 3H), 1.86 (dpd, *J* = 13.4, 6.7, 2.0 Hz, 8H), 1.69–1.58 (m, 3H), 1.58–1.38 (m, 6H), 1.05–0.83 (m, 46H), 0.65–0.56 (m, 14H) ppm. ^13^C NMR (101 MHz, CDCl_3_): δ, 220.9, 172.2, 148.7, 138.1, 137.4, 128.2, 126.7, 126.5, 125.3, 124.8, 121.7, 121.7, 118.9, 118.8, 50.6, 48.1, 44.3, 38.1, 36.0, 34.7, 34.5, 31.7, 29.5, 28.4, 26.5, 25.9, 25.8, 24.0, 23.9, 23.9, 22.7, 22.6, 22.6, 22.5, 21.7, 18.2, 13.9, 1.2 ppm (Supplementary Materials). **IR** (film, DCM) ν 3455, 3077, 2952, 2928, 2870, 1739, 1641, 1608, 1583, 1494, 1453, 1417, 1369, 1335, 1260, 1226, 1114, 1009, 915. **MS (TOF ES)**: *m/z* [M + Na]^+^: 1204.44

## 4. Conclusions

In summary, we explored the ruthenium-catalyzed cross metathesis reactions between allyl-POSS and five alkenes, including three steroid derivatives. We showed that, even for such challenging partners and relatively mild conditions, such as 0.5–2 mol% of catalyst at DCM or toluene at RT to 100 °C, are sufficient to perform an efficient CM reaction with POSS derivatives. The best catalysts for these transformations were **Hov-II** and **Nitro** complexes.

## Figures and Tables

**Table 1:**
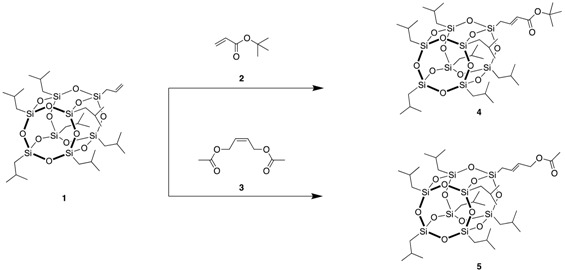
Cross metathesis of allyl-polyhedral oligomeric silsesquioxanes (POSS) derivative with model partners.

Entry	Catalyst	Time (h)	Yield of 4 (%)	Yield of 5 (%)
1	[Ru]-1	5	6	33
2	[Ru]-2	5	5	40
3	Gr-II	5	71	80
4	[Ru]-3	5	28	31
5	[Ru]-4	5	79	83
6	Hov-II	5	99	98
7	Hov-II	2	80	73
8	[Ru]-5	5	99	69
9	[Ru]-6	5	40	57
10	Ind-II	5	0	0
11	[Ru]-7	5	50	52
12	[Ru]-8	5	0	0
13	Nitro	24	90	64
14	Nitro	5	87	76
15	[Ru]-9	5	94	89
16	[Ru]-9	2	81	69
17	[Ru]-10	5	99	66
18	[Ru]-10	2	72	26
19	[Ru]-11	5	56	77
20	[Ru]-12	5	91	82
21	[Ru]-12	2	93	90
22	[Ru]-13	5	91	82
23	[Ru]-14	5	91	95
24	[Ru]-14	2	87	80
25	[Ru]-15	5	85	83
26	[Ru]-16	5	81	86
27	[Ru]-17	5	96	82
28	[Ru]-18	5	90	82

Reaction conditions: 1 (0.5 mmol), 2 (1 mmol), or 3 (1.5 mmol), [Ru] 0.5 mol%, dichloromethane (DCM) (4 mL), room temperature (RT).

**Table 2:**
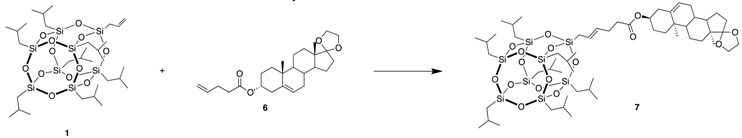
Cross metathesis of allyl-POSS **1** with derivative of androsterone **6**.

Catalyst (mol%)	Solvent	Temperature (°C)	Time (h)	Yield of 7 (%)
Hov-II (0.5)	DCM	RT	5	3
Hov-II (2)	DCM	45	5	36
Hov-II (2)	Toluene	100	5	69
Nitro (2)	Toluene	100	5	72

**Table 3:**
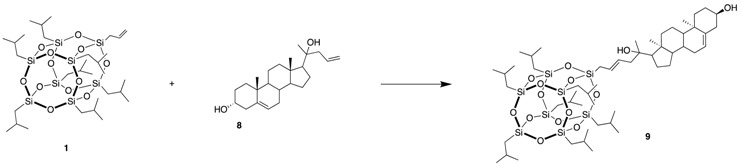
Cross metathesis of allyl-POSS **1** with derivative of pregnenolone **8**.

Catalyst (mol%)	Solvent	Temperature (°C)	Time (h)	Yield of 9 (%)
Hov-II (0.5)	DCM	RT	5	29
Hov-II (2)	Toluene	100	5	36
Nitro (2)	Toluene	100	10	46
Nitro (2)	DCM	45	24	69

## Supplementary Materials

The following are available online. Figure S1: ^1^H NMR of compound **4**; Figure S2: ^13^C NMR of compound **4**; Figure S3: ^1^H NMR of compound **5**; Figure S4: ^13^C NMR of compound **5**; Figure S5: ^1^H NMR of compound **7**; Figure S6: ^13^C NMR of compound **7**; Figure S7: ^1^H NMR of compound **9**; Figure S8: ^13^C NMR of compound **9**; Figure S9: ^1^H NMR of compound **11**; Figure S10: ^1^H NMR of compound **11**.
