# Supplementary material for: Modification of Polyhedral Oligomeric Silsesquioxanes (POSS) Molecules by Ruthenium Catalyzed Cross Metathesis
Source: Molecules. 2018 Jul 14;23(7):1722. doi: 10.3390/molecules23071722 (PMC6099925; doi:10.3390/molecules23071722)
Supplement: Supplementary file 1 [file molecules-23-01722-s001.pdf]

Supporting Information

# Modification of POSS nanoparticles by Ruthenium Catalyzed Cross metathesis

Justyna Czaban-Jóźwiak,[1‡] Łukasz Woźniak,[1] Artur Ulikowski,[1] Katarzyna Kwiecińska,[1] Adam Rajkiewicz,[1] Karol Grela*[1]

[1]Institute of Organic Chemistry, Polish Academy of Sciences, Kasprzaka 44/52, PO Box 58, Warsaw, Poland karol.grela@gmail.com http://www.karolgrela.eu/ +48-22-632-66-81

[‡] Current address: Advanced Membranes & Porous Materials Center, Division of Physical Sciences and Engineering, Functional Materials Design, King Abdullah University of Science and Technology, Thuwal, Saudi Arabia

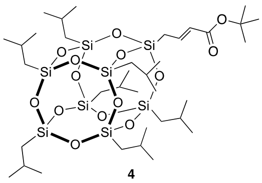


**4**

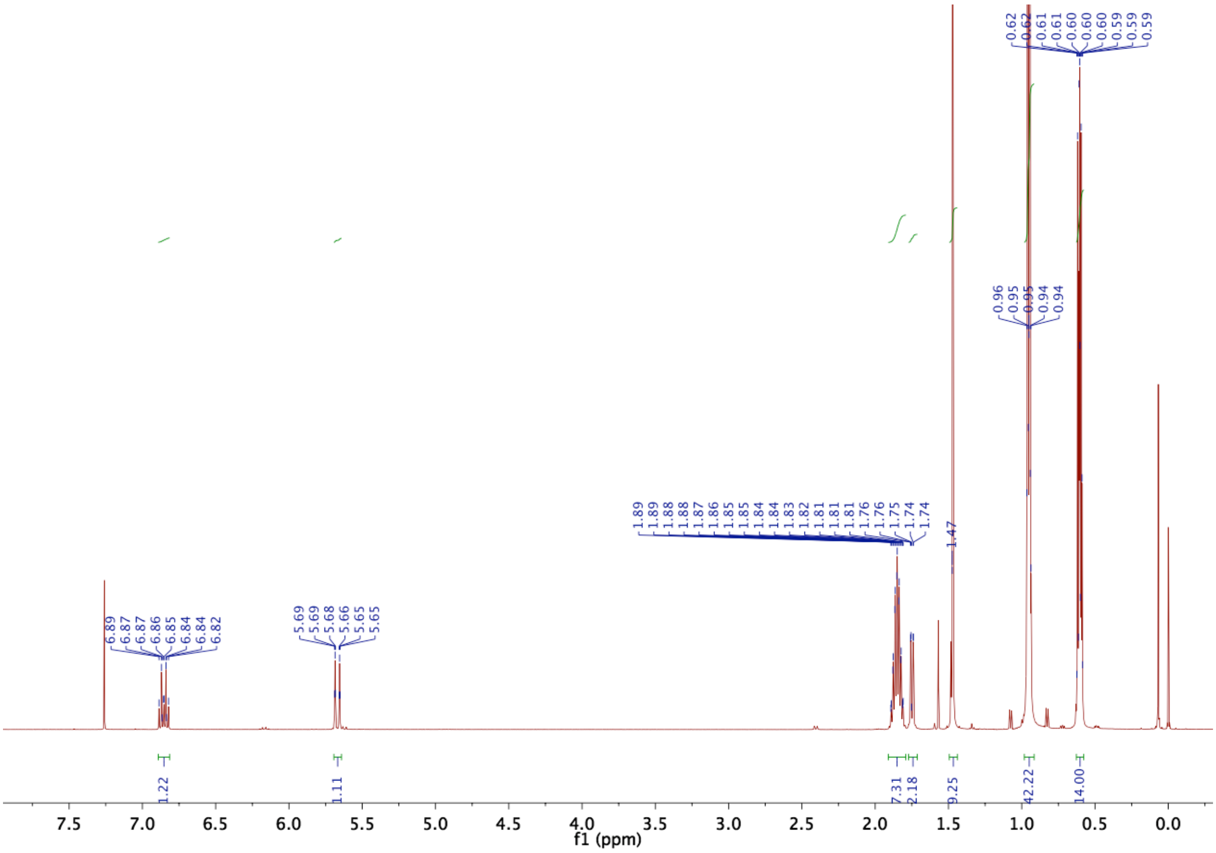


Figure 1. $^{1}$H NMR of compound **4**

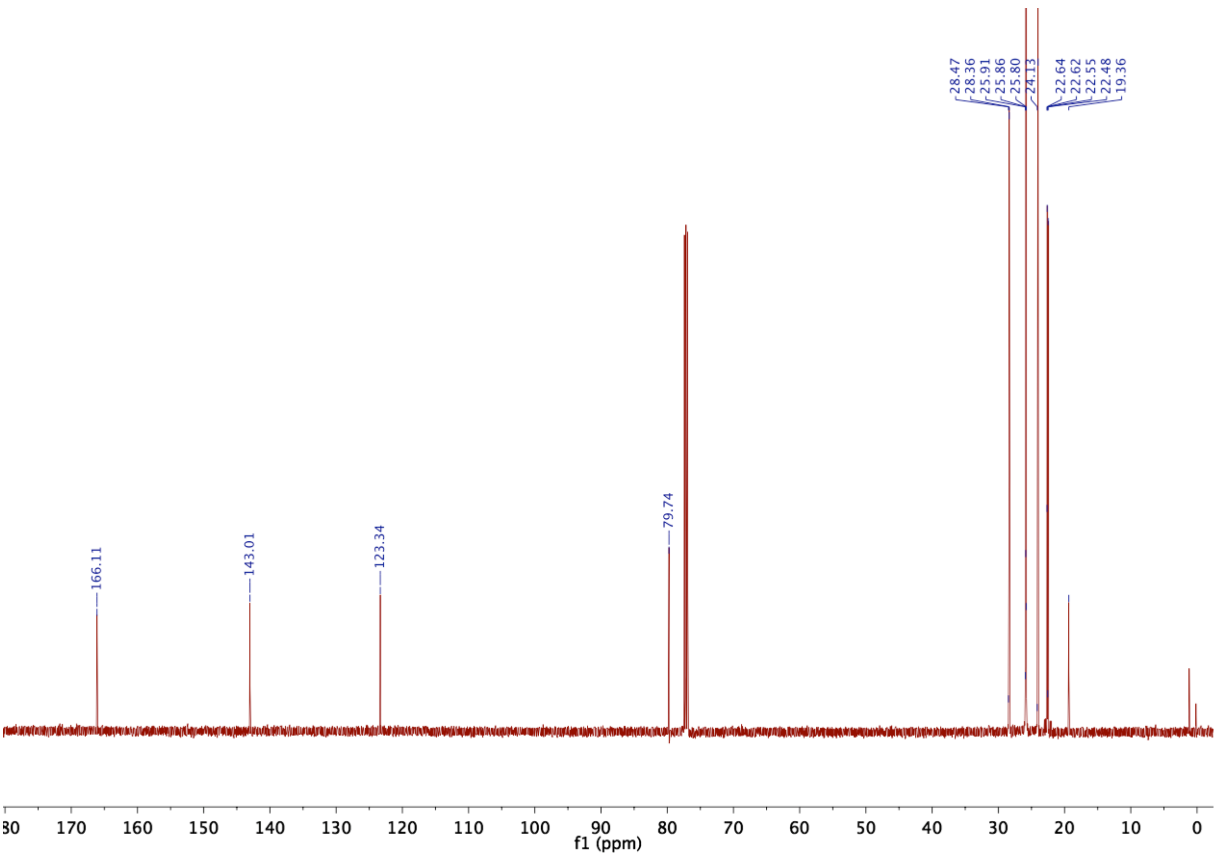


Figure 2. $^{13}C$ NMR of compound **4**

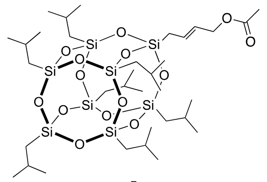

5

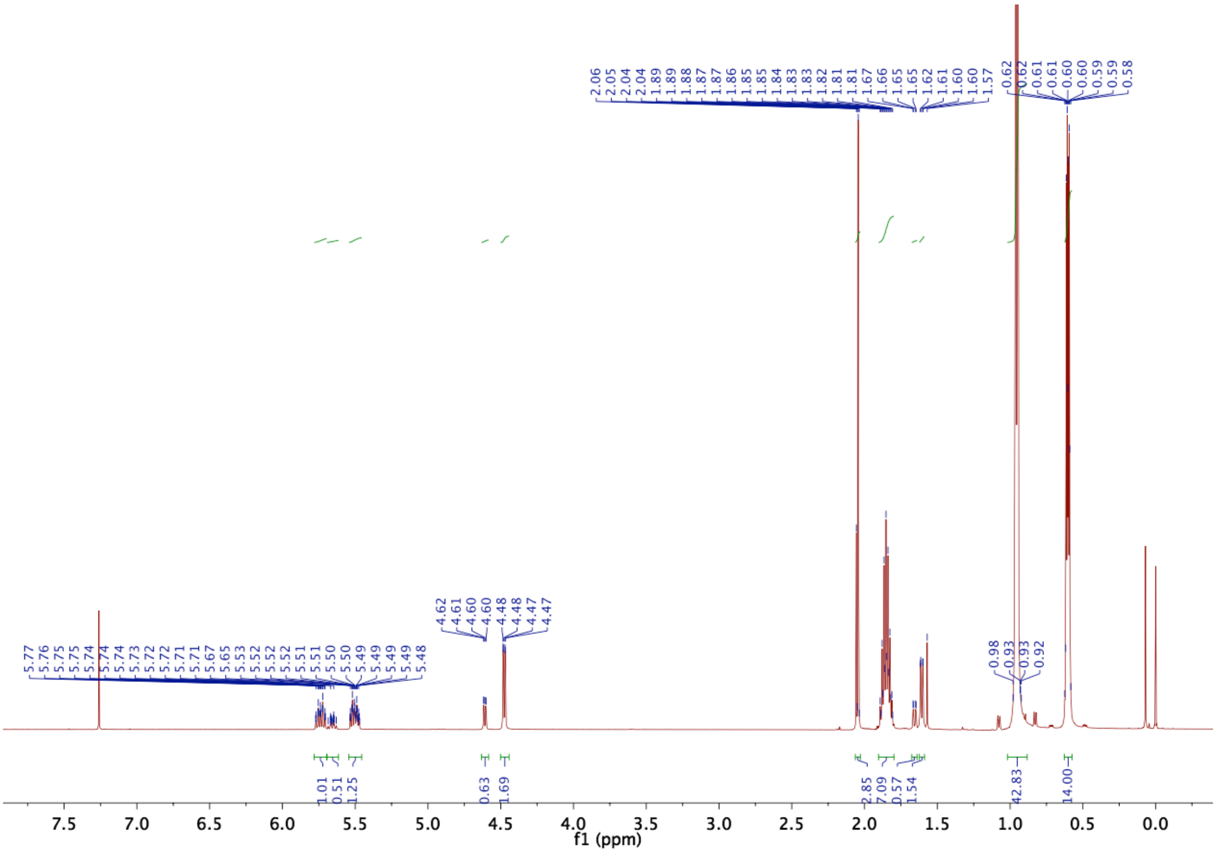

Figure 3. $^{1}$H NMR of compound **5**

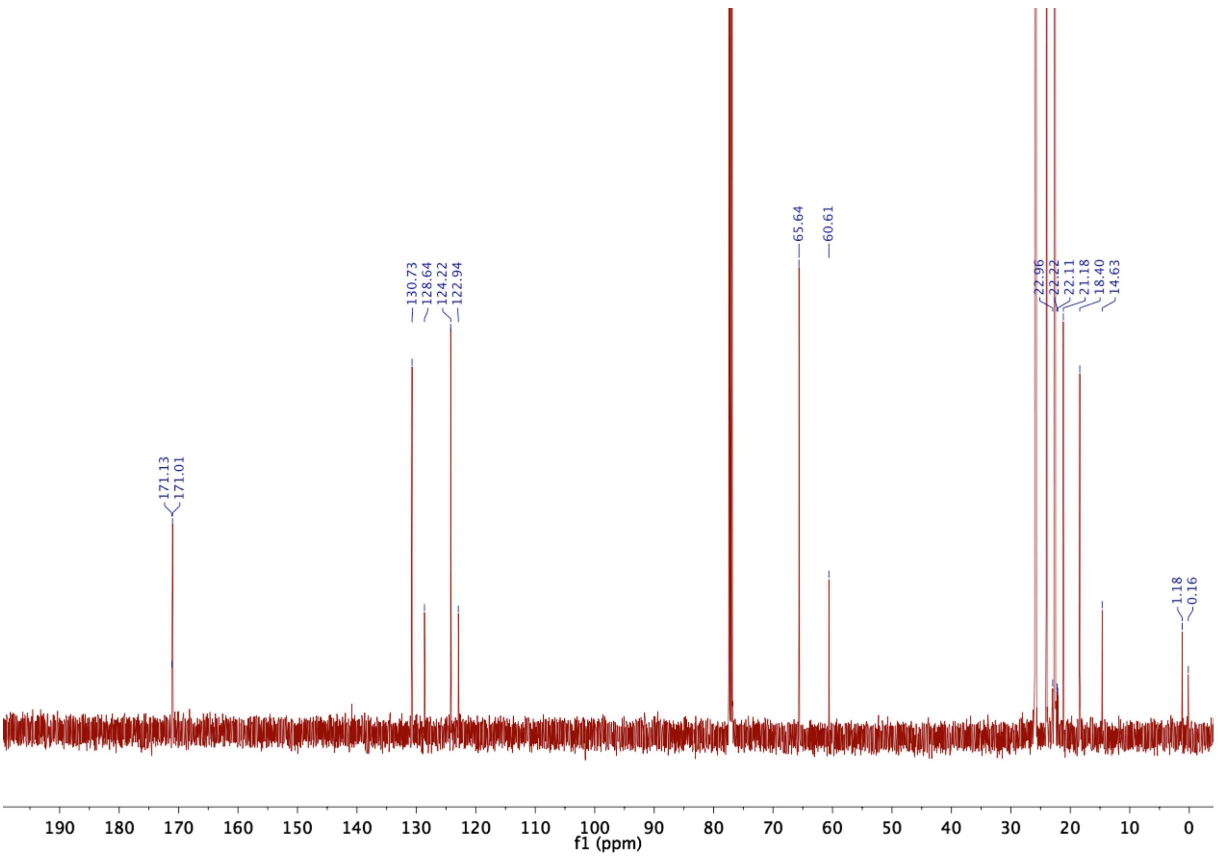


Figure 4. $^{13}C$ NMR of compound **5**

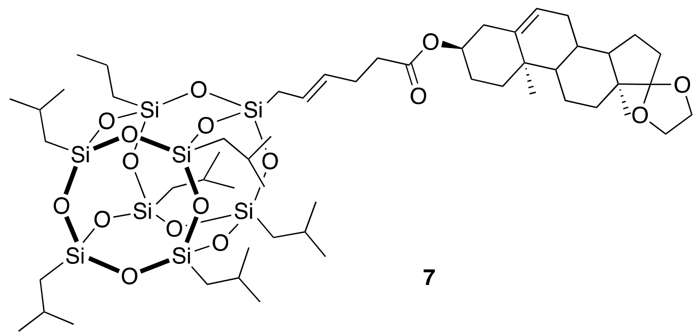

**7**

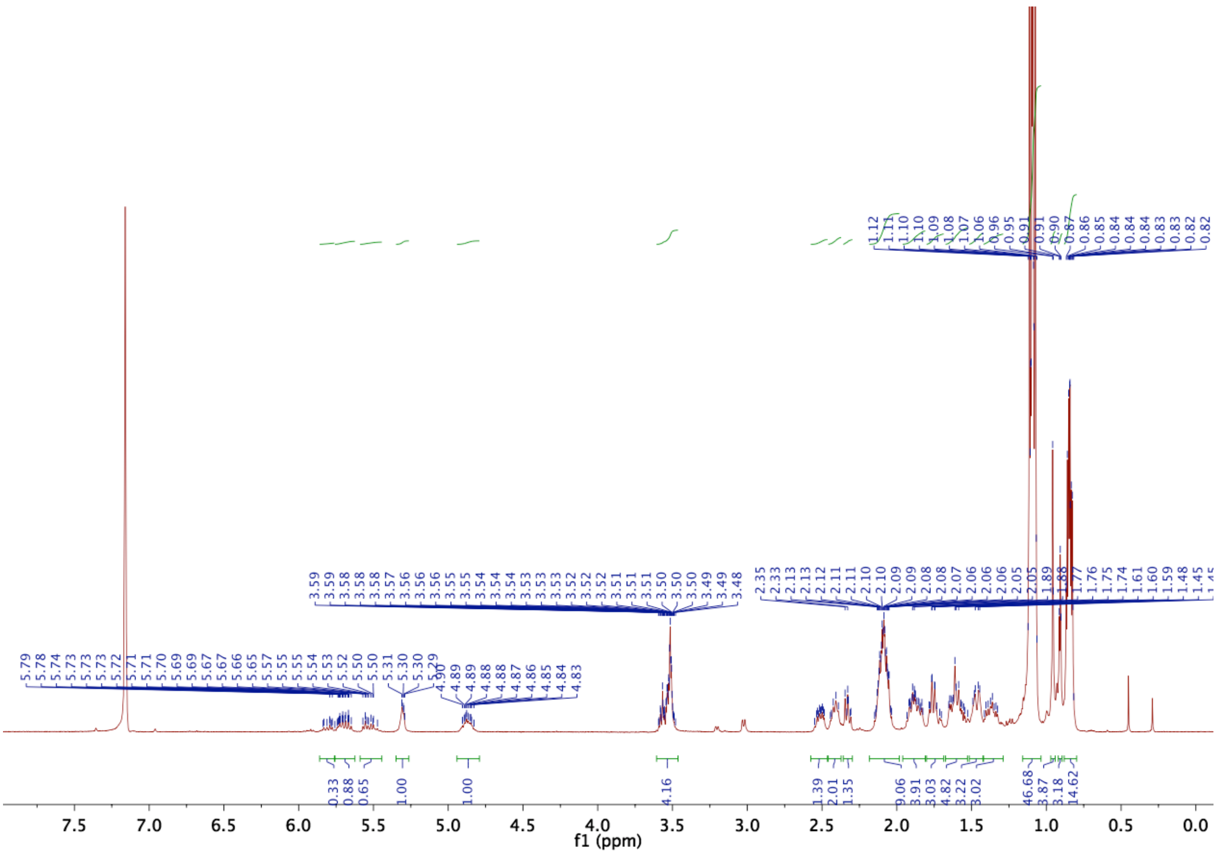


Figure 5. $^{1}$H NMR of compound **7**

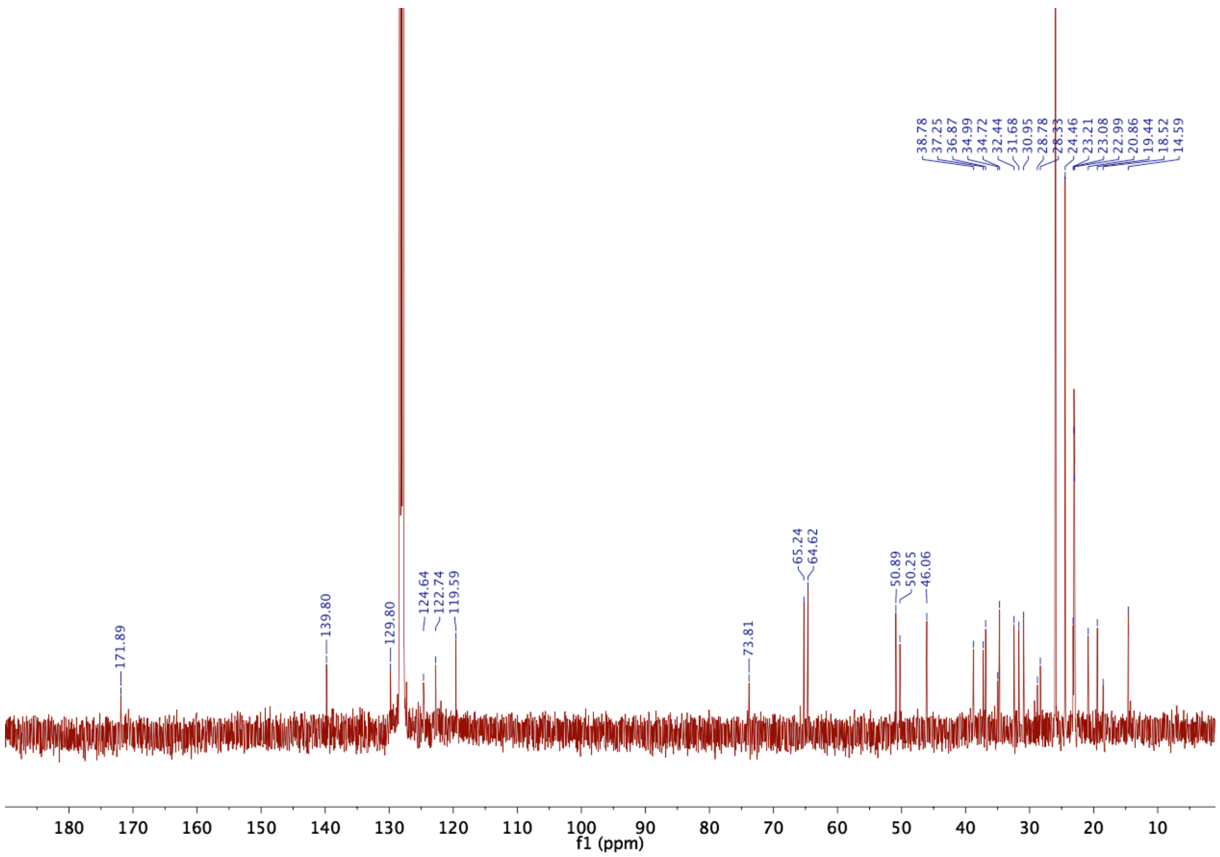


Figure 6. $^{13}C$ NMR of compound **7**

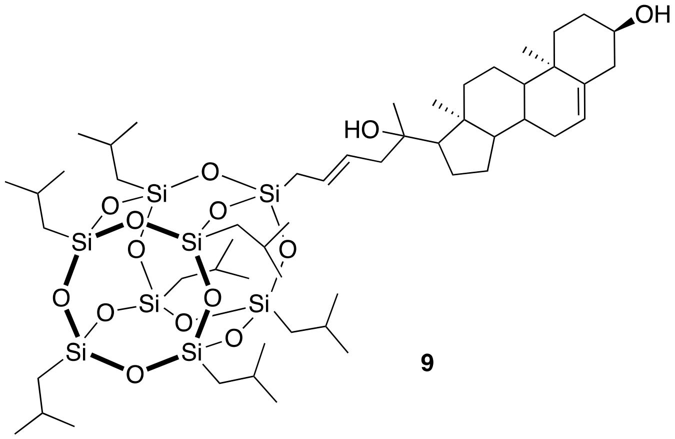


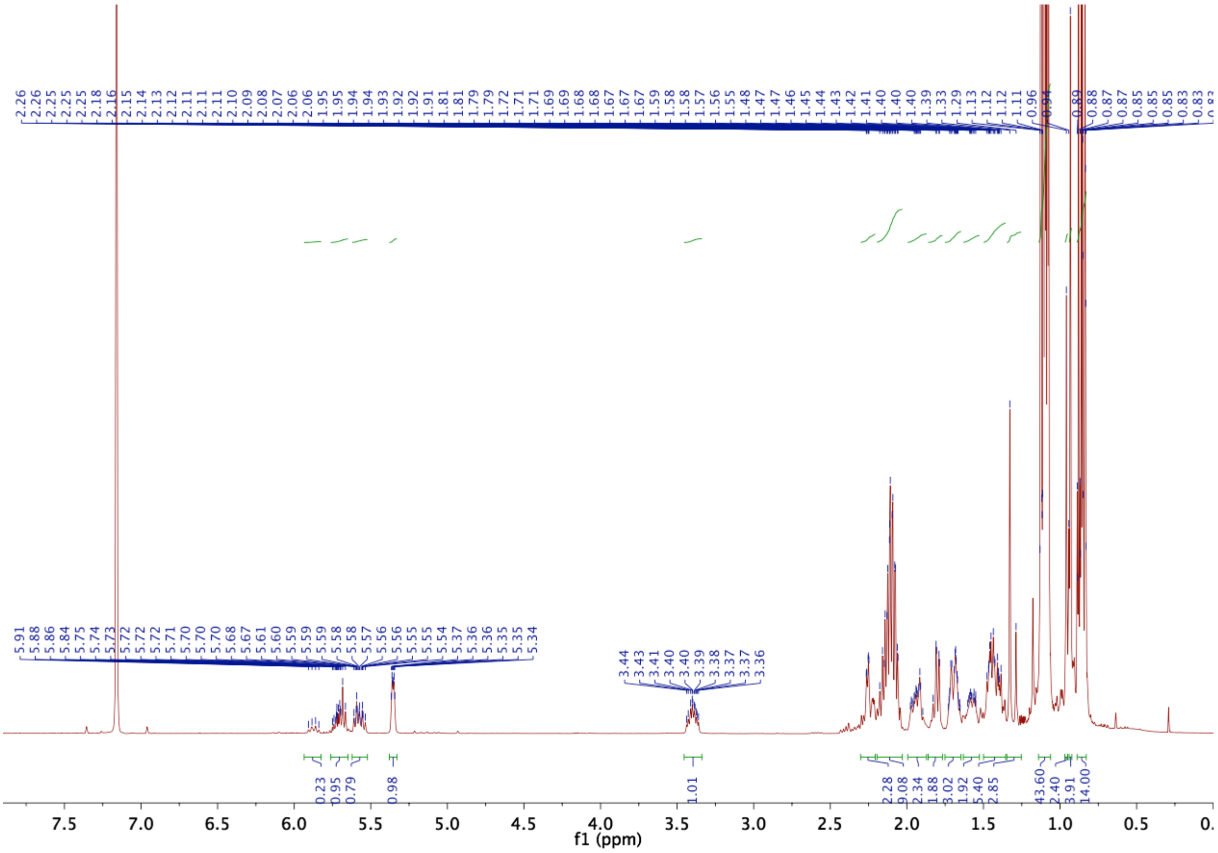


Figure 7. $^{1}$H NMR of compound **9**

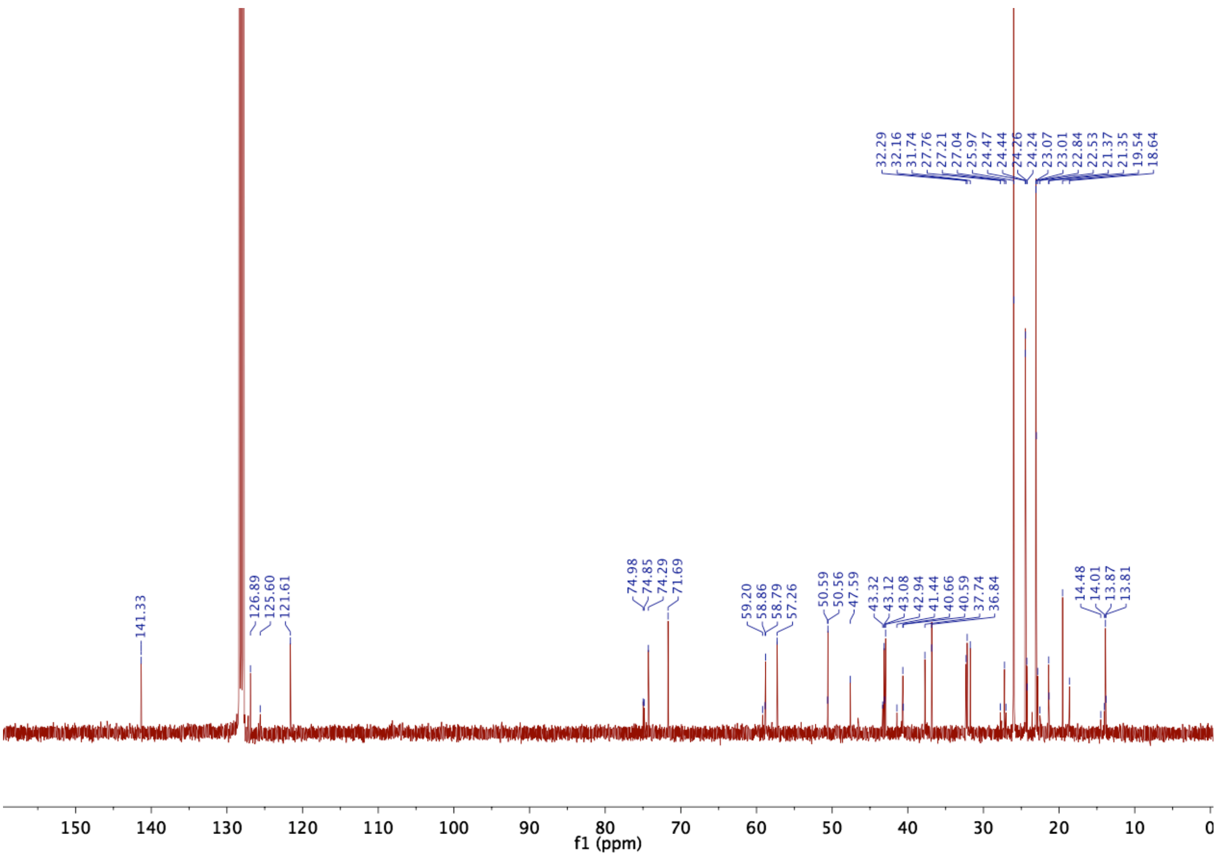


Figure 8. $^{13}$C NMR of compound **9**

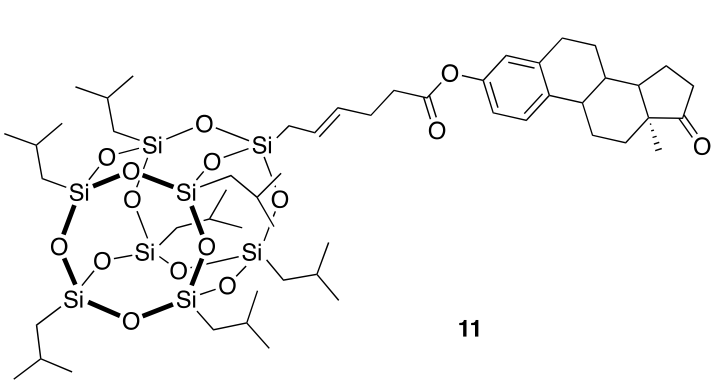

**11**

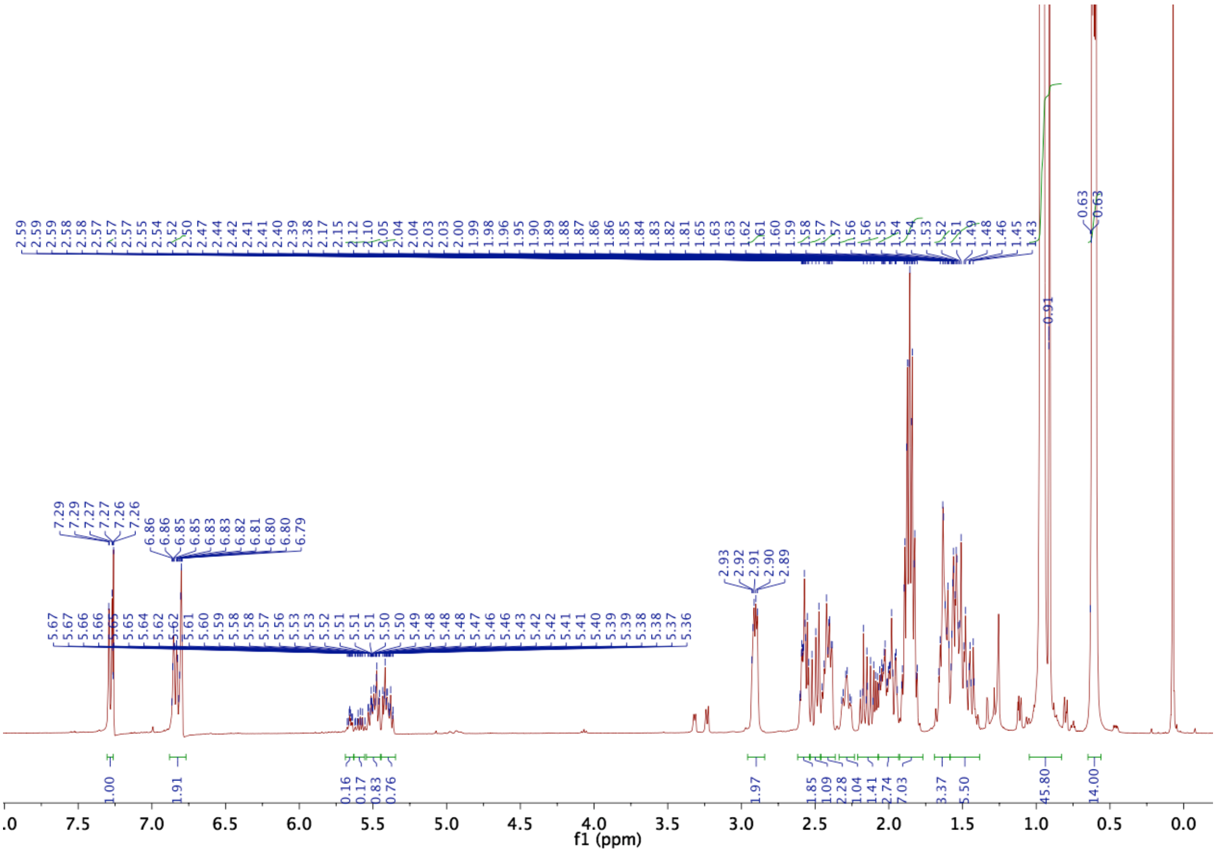

Figure 9. $^{1}H$ NMR of compound **11**

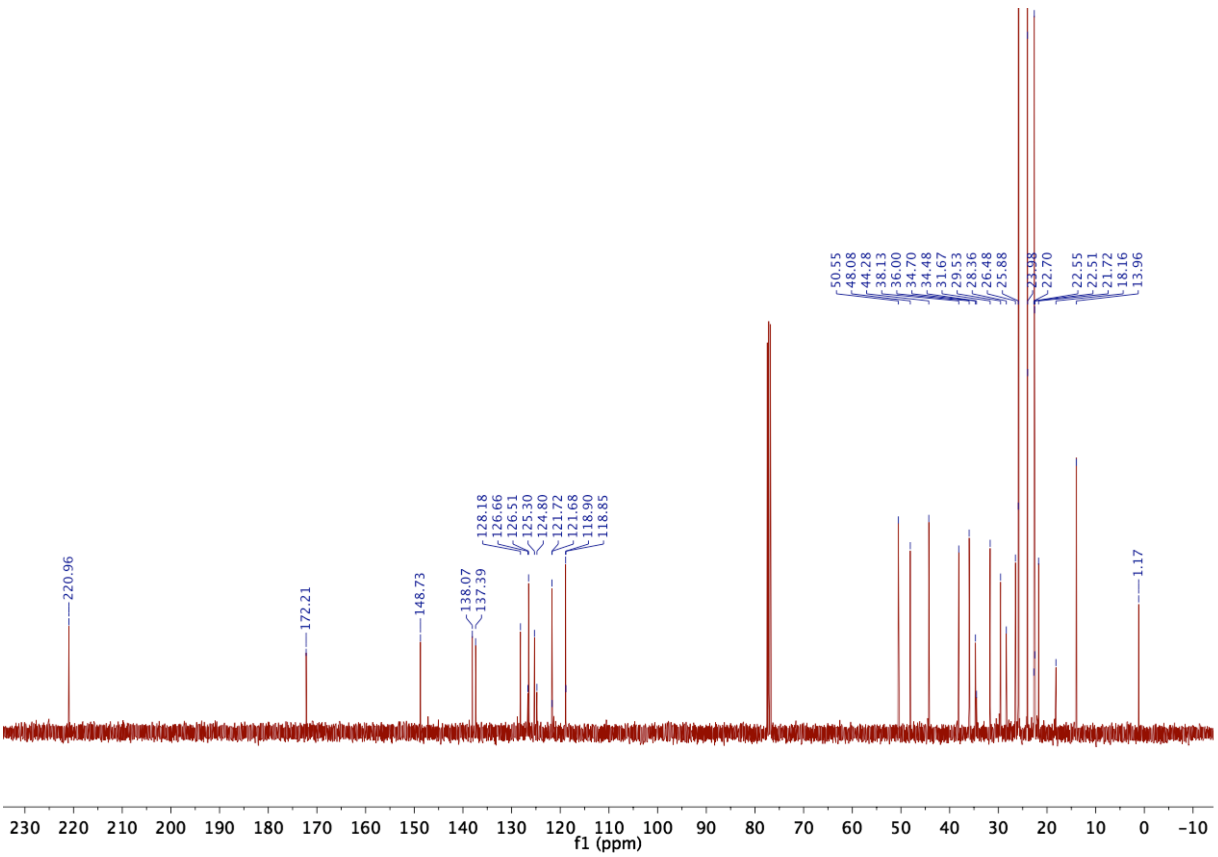


Figure 10. $^{1}$H NMR of compound **11**
